# Heterogeneity and associated factors of palliative care core competency among oncology nurses: a latent profile analysis

**DOI:** 10.7717/peerj.21645

**Published:** 2026-08-26

**Authors:** Yang Yu, Fangxin Luoye, Danhui You, Zhenhong Xu, Yuping Liu, Li Wu, Qian Deng, Yanting Ning, Liqiong Liu

**Affiliations:** 1 Medical Oncology, National Cancer Center/National Clinical Research Center for Cancer/Cancer Hospital & Shenzhen Hospital, Chinese Academy of Medical Sciences and Peking Union Medical College, Shenzhen, Guangdong, China; 2 Department of Nursing, National Cancer Center/National Clinical Research Center for Cancer/Cancer Hospital & Shenzhen Hospital, Chinese Academy of Medical Sciences and Peking Union Medical College, Shenzhen, Guangdong, China; 3 Department of Radiation Oncology, National Cancer Center/National Clinical Research Center for Cancer/ Cancer Hospital & Shenzhen Hospital, Chinese Academy of Medical Sciences and Peking Union Medical College, Shenzhen, Guangdong, China

**Keywords:** Palliative care, Oncology nurse, Core competency, Latent profile analysis

## Abstract

**Background:**

Global cancer incidence is expected to rise by 60% by 2040, intensifying the burden experienced by patients and their families. Palliative care improves quality of life in advanced disease and depends on oncology nurses’ competencies in ethical decision-making, symptom management, and psychological resilience. Despite moderate overall proficiency, psychosocial shortcomings and heterogeneity in competency remain insufficiently characterized. This study utilized latent profile analysis (LPA), guided by shared decision-making and symptom management theories, to identify competency subgroups and associated factors among Chinese oncology nurses, aiming to guide targeted training interventions.

**Methods:**

A cross-sectional survey was conducted among 241 oncology nurses from five hospitals in Shenzhen, China, between April and September 2025, using cluster sampling. Data were collected using a general information questionnaire, the 32-item Core Competency Questionnaire, and the Nurses’ Professional Identity Scale. LPA models (1–4 classes) were fitted using R software (version 4.4.0). Univariate analyses, Analysis of Variance or Kruskal–Wallis H tests, and multivariate logistic regression were performed with SPSS 29.0 to evaluate demographic and occupational factors associated with profile membership.

**Results:**

The mean core competency score was 76.02 ± 23.85 (59.3% of maximum). LPA revealed three distinct profiles: low-competency (27%), moderate -competency (45%), and high-competency (28%). The three-profile solution was selected by considering model fit indices, entropy, class proportions, and substantive interpretability (entropy = 0.931; Lo-Mendell-Rubin and bootstrap likelihood ratio tests *P* < 0.001). High-competency nurses scored 1.8-fold higher in ethical care, 2.4-fold higher in clinical practice, and 2.0-fold higher in psychological self-adjustment compared with the low-competency group (all *P* < 0.001). Systematic palliative care training was associated with higher odds of membership in the high-competency profile rather than the moderate-competency profile (*OR* = 3.210, 95% CI [1.298–7.940], *P* = 0.012). Higher average monthly night-shift frequency was associated with lower odds of membership in the low-competency profile rather than the moderate-competency profile (*OR* = 0.887, 95% CI [0.788–0.998], *P* = 0.046). Profiles diverged significantly in professional identity (F = 24.11, *P* < 0.001, *η*^2^ = 0.17).

**Conclusion:**

Oncology nurses exhibit heterogeneous palliative care competencies characterized by strong ethical, moderate clinical, and weak psychological capacities. Systematic training was associated with high-competency profile membership, and targeted programs focusing on psychological self-adjustment may be explored in future longitudinal or interventional studies.

## Introduction

Globally, cancer accounts for approximately 10 million deaths annually, with projections indicating a substantial increase in incidence by 2040, highlighting its scale as a public health challenge (Bray et al., 2024). Palliative care improves quality of life in advanced cancer through multidimensional interventions (Hart et al., 2022; Han et al., 2024). One important question is whether oncology nurses have enough skills in ethical decision making, symptoms management and psychological adjustment to support these interventions (Zhang et al., 2024). China population ageing and increasing cancer burden are increasing the demand for high quality palliative care. Oncology nurses are the main professionals involved in end-of-life care in cancer-related clinical settings and the national policies emphasize more and more the development of palliative care services (Zhao et al., 2024).

Palliative care incorporates multidisciplinary strategies to address physical, emotional, and existential distress in life-limiting illnesses, thereby substantially enhancing quality of life and alleviating healthcare burdens (Woo & Lee, 2023; Sharratt et al., 2024). Amid an aging population and increasing cancer burden, the quality of palliative care services has become an important indicator of healthcare system performance. As the core providers of this service, nurses’ professional core competency directly affects the quality of care and patient experience. Therefore, enhancing oncology nurses’ core competency in palliative care is of considerable clinical and public health importance (Li et al., 2023).

Core competencies in palliative care are inherently multidimensional, encompassing ethical decision-making, symptom management, and psychological support. These dimensions are reflected in existing theoretical perspectives, such as shared decision-making (Barry & Edgman-Levitan, 2012) and symptom management frameworks (Silva, Lopes & Mercês, 2021), which together highlight the complexity of nursing roles in end-of-life care. Specifically, shared decision-making emphasizes communication, value clarification and patient participation in preference-sensitive care decisions closely related to ethical care competency in palliative care. Symptom management frameworks focus on symptom assessment, intervention, and outcome evaluation, related to clinical practice competence and psychological adjustment in advanced cancer care. These perspectives provide a conceptual basis for understanding palliative care competency as a multidimensional construct rather than a single overall skill. However, current research has rarely translated these conceptual insights into empirical investigations of competency structures among oncology nurses.

Existing international studies have progressively moved from defining competency dimensions and developing assessment tools to evaluating training effectiveness (Nouhi, Faramarzpour & Shahrbabaki, 2021). Recent research has shifted focus from overall average competency scores to identifying distinct nurse subgroups with varying competency profiles across ethical, clinical, and psychological dimensions (Tiantian et al., 2025).

For instance, Nouhi, Faramarzpour & Shahrbabaki (2021) reported that oncology nurses exhibit imbalanced competencies in symptom management (clinical practice) *versus* bereavement counseling (psychological support) dimensions; similarly, the Lunder team used distinct nurse profiles with varied competency strengths through cluster analysis (Paatela et al., 2024). In China, although research emerged later, it has advanced swiftly, focusing on status assessments, factor analyses, and training explorations (Tiantian et al., 2025). A consensus emerges that Chinese nurses’ palliative care competency remains at an overall moderate level, exhibiting pronounced shortfalls in psychosocial support and ethical practice.

However, a literature review reveals two significant gaps in current research: first, most existing studies have used variable-centered approaches, such as comparisons of mean scores or regression analyses. Although these methods are useful for identifying overall levels and associated factors, they usually assume that nurses constitute a relatively homogeneous population. Such approaches may obscure distinct subgroups of nurses who have different strengths and weaknesses across ethical care, clinical practice, and psychological self-adjustment. Second, although shared decision-making and symptom management theories are highly relevant to oncology nurses’ palliative care practice, few studies have used these frameworks to guide person-centered analyses of competency heterogeneity.

To address these gaps, this study applies latent profile analysis (LPA), a person-centered approach that identifies latent subgroups based on multidimensional data. Compared with conventional variable-centered methods, LPA can classify individuals into unobserved profiles according to their response patterns across multiple competency dimensions. This method is suitable for identifying whether oncology nurses share similar or divergent patterns of ethical care, clinical practice, and psychological self-adjustment. By integrating multidimensional competency constructs derived from existing theoretical perspectives, this study aims to explore latent profiles of palliative care core competency among oncology nurses in specialized cancer hospitals and to analyze their influencing factors. This approach may provide evidence for developing targeted training and management strategies.

## Methods

### Study design

This cross-sectional study recruited oncology nurses from five hospitals in Shenzhen from April to September 2025. We used the Strengthening the Reporting of Observational Studies in Epidemiology (STROBE) statement guidelines (Von Elm et al., 2007) to report this study (File S1). The inclusion criteria were possession of a valid nurse practicing certificate and at least one year of work experience in an oncology-related clinical setting. All voluntary participants signed written informed consent forms and were informed of the research purpose and anonymity safeguards prior to the survey. The exclusion criteria included nurses who were engaged in advanced studies, who were on personal leave, or who were on medical leave. Cluster sampling was used in the recruitment process. The participating hospitals were treated as natural clusters, and eligible nurses from oncology-related departments within these institutions were invited to participate according to the inclusion and exclusion criteria.

Specifically, given the multicenter design of this study, hospitals were treated as natural clusters, allowing efficient access to oncology nurses within each institution. This approach not only improved feasibility and cost-effectiveness but also increased the diversity of the sample across different hospital settings in Shenzhen. However, because the participating hospitals were all located in Shenzhen, the sample should be interpreted as a regional multicenter sample rather than a nationally representative sample. For latent profile analysis, there is no universally accepted fixed rule for determining sample size. Therefore, sample adequacy was considered in relation to the number of profile indicators, model convergence, profile size, entropy, statistical fit indices, and interpretability of the final solution (MacCallum et al., 2001). In this study, 241 valid questionnaires were included, and the smallest latent profile contained 65 participants, which supported the feasibility of conducting LPA with three continuous competency indicators. A total of 300 questionnaires were distributed, with 241 valid responses returned, yielding a valid response rate of 80.3%.

### Measurements

A self-designed general information questionnaire captured demographic and occupational variables, including age, gender, education level, working experience, professional title, palliative care training history, and experience caring for terminal-stage patients.

### Nurse palliative care core competency questionnaire

The Palliative Care Nurse Core Competency Questionnaire, developed by Han (2021) was selected. This questionnaire comprises three dimensions: ethical care competency (ECC), clinical practice competency (CPC), and psychological self-adjustment ability (PSA), for a total of 32 items. This self-assessment questionnaire employs a Likert 5-point scoring system: 0 points represent ‘no ability’, 1 point represents ‘minimal ability’, 2 points represent ‘some ability’, 3 points represent ‘sufficient ability’, and 4 points represent ‘strong ability’. The total score ranges from 0 to 128 points, with higher scores indicating stronger core competency in palliative care among nurses. The content validity index (S-CVI/Ave) of this questionnaire was 0.911, with a Cronbach’s α of 0.980, a split-half reliability of 0.907, and a test-retest reliability of 0.959, demonstrating good reliability and validity.

### Nurse professional identity scale

The Nurses’ Professional Identity Scale was developed by Liu, (2009) to assess nurses’ level of professional identity. It contains 30 items across five dimensions: professional cognitive evaluation (nine items), professional social support (six items), professional social skills (six items), professional frustration coping (six items), and professional self-reflection (three items). A 5-point Likert scale was used, with responses ranging from ‘Strongly Disagree’ (scored as 1) to ‘Strongly Agree’ (scored as 5). Total scores range from 30–150, with higher scores indicating a higher level of professional identity. This scale has been demonstrated to have good reliability and validity, with a Cronbach’s α of 0.891.

### Data collection

Trained researchers distributed questionnaires onsite, providing instructions on study purpose and completion guidelines. The questionnaires were completed anonymously and collected onsite. Post-collection, data were screened for completeness and logical consistency, excluding invalid responses. Questionnaires with missing key variables, duplicated responses, or logically inconsistent answers were excluded before analysis. The final analysis was therefore based on complete valid questionnaires, and no statistical imputation was performed for invalid or incomplete questionnaires.

### Statistical methods

Data analysis utilized R (version 4.4.0) for LPA and SPSS (version 29.0; IBM Corp., Armonk, NY, USA) for descriptives and inferential tests. LPA is a model-based clustering method that clarifies relationships among exogenous continuous indicators through hypothesized categorical variables while maintaining local independence between these indicators. The three dimension scores of palliative care core competency, namely ECC, CPC, and PSA, were used as continuous indicators in the LPA. The assumptions considered in the LPA included the use of continuous profile indicators, local independence of indicators within each latent profile, model convergence, adequate profile size, and substantive interpretability of the extracted profiles. Models (1–4 classes) were fitted using log-likelihood, Akaike information criterion (AIC), Bayesian information criterion (BIC), adjusted BIC (aBIC), entropy, Lo-Mendell-Rubin likelihood ratio test (LMR), and bootstrap likelihood ratio test (BLRT). Lower AIC/BIC/aBIC values denoted parsimonious fit; LMR/BLRT *P* <0.050 indicated superior k-class models; entropy > 0.80 signified clear profile separation. The final profile solution was selected by considering statistical fit indices, entropy, profile proportions, theoretical meaningfulness, and interpretability rather than relying on a single fit index.

SPSS 29.0 was utilized for preliminary data processing and descriptive statistical analysis. Categorical data are presented as frequencies and percentages. Continuous data are presented as the means and standard deviations when normality was confirmed by Kolmogorov-Smirnov tests. Nonnormally distributed data are presented as medians with interquartile ranges (M) (P25–P75). When intergroup differences were compared, chi-square tests or Fisher’s exact tests were employed for categorical variables. For continuous variables, either analysis of variance (ANOVA) or the Kruskal–Wallis H test was used on the basis of data normality. Additionally, multivariate logistic regression models were utilized to assess differences in demographic characteristics and determine their predictive effects on latent profile membership. Latent profile membership was used as the dependent variable, with the moderate-competency group set as the reference category. Variables with statistical significance in univariate analyses and theoretically relevant covariates were considered for inclusion in the multivariate logistic regression model. Before model fitting, multicollinearity among candidate predictors was assessed using tolerance and variance inflation factor (VIF) values. Statistical significance was set at *P* < 0.050. The internal consistency of the scale was evaluated using the Cronbach’s alpha coefficient and the McDonald’s omega coefficient. The McDonald’s omega coefficient is regarded as a more accurate reliability assessment indicator because it does not assume tau equivalence.

### Ethical approval

This study obtained approval from the Institutional Review Board of the National Cancer Center/National Clinical Research Center for Cancer/Cancer Hospital, Chinese Academy of Medical Sciences and Peking Union Medical College, along with Shenzhen Hospital (Approval No. KYKT2025-11-1), in compliance with the ethical principles of the Declaration of Helsinki. All participants provided electronic informed consent and retained the right to withdraw from the study at any time without affecting subsequent treatment.

## Result

### Characteristics of the study subjects

This study included 241 nurses from five hospitals in Shenzhen, with 96.7% being female. The mean age was 34.2 ± 6.8 years, and 90.9% held a bachelor’s degree or higher. Among them, 63.9% had received palliative care training, 18.7% had engaged in related work for more than 12 months, and 86.7% had cared for patients in the terminal stage. Univariate analysis revealed significant differences in the total score of core competency based on sex, hospital classification, duration of palliative care work, palliative care training experience, and bereavement experience within the past year (*P* < 0.050) (Table 1).

**Table 1:** Univariate analysis of general characteristics and palliative care core competency scores (*N* = 241).

**Variable**	**N (%)**	**Total score** **(mean ± SD)**	**t/F value**	***P*** ***value***
**Age**			1.021	0.362
20–30 years	83 (34.4)	78.77 ± 26.04		
30–40 years	114 (47.3)	73.87 ± 22.93		
Over 40 years	44 (18.3)	76.39 ± 21.79		
**Gender**			−2.815	**0.005**
Male	8 (3.3)	53.00 ± 15.66		
Female	233 (96.7)	76.81 ± 23.71		
**Education level**			0.988	0.399
Junior college or below	22 (9.1)	69.55 ± 25.55		
Bachelor’s degree	207 (85.9)	76.47 ± 23.68		
Master’s degree	11 (4.6)	78.09 ± 23.74		
Doctoral degree	1 (0.4)	102.00 ± 0.00		
**Marital status**			0.194	0.824
Unmarried	83 (34.4)	75.95 ± 26.89		
Married	156 (64.7)	75.92 ± 22.24		
Divorced or widowed	2 (0.8)	86.50 ± 19.09		
**Ethnicity**			−0.202	0.840
Han Chinese	223 (92.5)	75.93 ± 23.91		
Other	18 (7.5)	77.11 ± 23.74		
**Religious affiliation**			0.789	0.431
None	234 (97.1)	76.23 ± 23.83		
Present	7 (2.9)	69.00 ± 25.57		
**Hospital classification**			6.015	**<0.001**
Tertiary hospital	182 (75.5)	79.24 ± 23.15		
Secondary hospital	22 (9.1)	59.82 ± 23.02		
Primary hospital	31 (12.9)	71.55 ± 23.70		
Senior care facility	6 (2.5)	60.83 ± 19.66		
**Department**			2.120	0.079
Palliative care unit	24 (10.0)	75.75 ± 22.84		
Oncology department	141 (58.5)	79.25 ± 23.03		
Traditional Chinese and Western medicine integration department	6 (2.5)	61.83 ± 35.79		
Geriatrics department	18 (7.5)	73.39 ± 23.30		
Other	52 (21.6)	69.92 ± 24.24		
**Average number of night shifts per month**			0.185	0.907
0–1 times	2 (0.8)	66.00 ± 5.66		
2–4 times	166 (68.9)	76.46 ± 24.20		
4–8 times	38 (15.8)	76.03 ± 22.63		
>8 times	35 (14.5)	74.46 ± 24.62		
**Working years**			0.281	0.755
<10 years	107 (44.4)	77.31 ± 26.17		
10–20 years	107 (44.4)	75.02 ± 22.01		
>20 years	27 (11.2)	74.85 ± 23.85		
**Palliative care working duration**			3.700	**0.012**
>12 months	45 (18.7)	83.98 ± 20.63		
3–12 months	68 (28.2)	73.68 ± 23.54		
1–3 months	35 (14.5)	81.77 ± 27.10		
0	93 (38.6)	71.71 ± 23.22		
**Average monthly income**			1.501	0.225
Below 3,000 yuan	3 (1.2)	95.33 ± 27.59		
3,000–5,000 yuan	28 (1.6)	80.00 ± 26.25		
Above 5,000 yuan	210 (87.1)	75.21 ± 23.43		
**Is monthly income within** **expected range**			1.352	0.178
Yes	140 (58.1)	77.78 ± 23.94		
No	101 (41.9)	73.57 ± 23.64		
**Professional title**			0.379	0.823
Nurse	31 (12.9)	78.71 ± 27.24		
Senior Nurse	82 (34.0)	77.74 ± 26.09		
Nurse-in-charge	112 (46.5)	74.19 ± 21.45		
Associate Chief Nurse	13 (5.4)	74.92 ± 23.49		
Chief Nurse	3 (1.2)	74.00 ± 17.52		
**Do you serve as a clinical instructor?**			0.604	0.546
Yes	100 (41.5)	77.12 ± 22.64		
No	141 (58.5)	75.23 ± 24.73		
**Have you received training in** **palliative care?**			2.381	**0.018**
Yes	154 (63.9)	78.74 ± 23.14		
No	87 (36.1)	71.20 ± 24.46		
**Have you experienced the death of a close family member in the past year?**			−2.528	**0.012**
Yes	47 (19.5)	68.21 ± 23.64		
No	194 (80.5)	77.91 ± 23.58		
**Have you cared for Patients in the terminal stage**			−0.219	0.827
Yes	209 (86.7)	76.15 ± 23.73		
No	32 (13.3)	75.16 ± 25.04		
**Your level of interest in palliative care work**			1.412	0.240
Very interested	50 (20.7)	80.62 ± 27.61		
Interested	121 (50.2)	76.40 ± 22.45		
General	67 (27.8)	71.69 ± 23.32		
Not interested	3 (1.2)	80.67 ± 14.36		
**Have you considered a career change**			0.875	0.382
Yes	61 (25.3)	78.33 ± 26.82		
No	180 (74.7)	75.23 ± 22.79		

**Notes.**

1. Data are presented as mean ± standard deviation for total scores and number (percentage) for categorical variables.

2. Group comparisons were performed using *t*-tests or one-way ANOVA, as appropriate.

3. Significant *P*-values are indicated in bold (*P* < 0.05, *P* < 0.01, *P* < 0.001).

4. Abbreviations: N, number of participants; %, percentage.

### Overall level of palliative care core competency

The mean total core competency score was 76.02 ± 23.85 (59.3% of maximum; range 0–128). Dimension scores were: ECC 22.9 ± 5.9 (71.6%), CPC 43.6 ± 15.9 (59.7%), and PSA 11.8 ± 3.9 (49.1%). PSA represented the lowest dimension.

### Latent profile analysis model fitting

One- to four-class LPA models were fitted (Table 2). The three-class model demonstrated the most appropriate overall solution: AIC = 4,592.695, BIC = 4,641.482, aBIC = 4,597.105, entropy = 0.931; LMR and BLRT *P* < 0. 001.The four-class model showed lower information criteria (AIC = 4,505.046, BIC = 4,567.772, aBIC = 4,510.716), but the LMR test was not significant (*P* = 0.061), and one latent class represented only 8.0% of the sample. Therefore, the three-class model was selected because it provided high classification accuracy, significant likelihood-ratio tests, adequate class proportions, and clearer substantive interpretability.

**Table 2:** Goodness-of-fit indices for latent subgroup models (*N* = 241).

Number of classes	Log(L)	AIC	BIC	aBIC	LMRT	BLRT	Entropy	K	Class proportion
1	−2,474.199	4,960.397	4,981.306	4,962.287				6	1
2	−2,329.941	4,679.882	4,714.73	4,683.032	<0.001	<0.001	0.822	10	0.510/0.490
3	−2,282.347	4,592.695	4,641.482	4,597.105	<0.001	<0.001	0. 931	14	0.270/0.450/0.280
4	−2,234.523	4,505.046	4,567.772	4,510.716	<0.061	<0.001	0.864	18	0.220/0.080/0.290/0.410

**Notes.**

1. Models with 1 to 4 latent classes were compared using multiple fit indices, including Akaike Information Criterion (AIC), Bayesian Information Criterion (BIC), adjusted BIC (aBIC), Lo-Mendell-Rubin Test (LMRT), Bootstrap Likelihood Ratio Test (BLRT), and entropy.

2. The 3-class model was selected as the optimal solution because it showed high entropy, significant LMRT and BLRT values, adequate class proportions, and clearer substantive interpretability. Although the 4-class model had lower information criteria, it was not selected because the LMRT was not significant (*P* = 0.061) and one class represented only 8.0% of the sample.

### Characteristics of latent profiles

**Figure 1:**
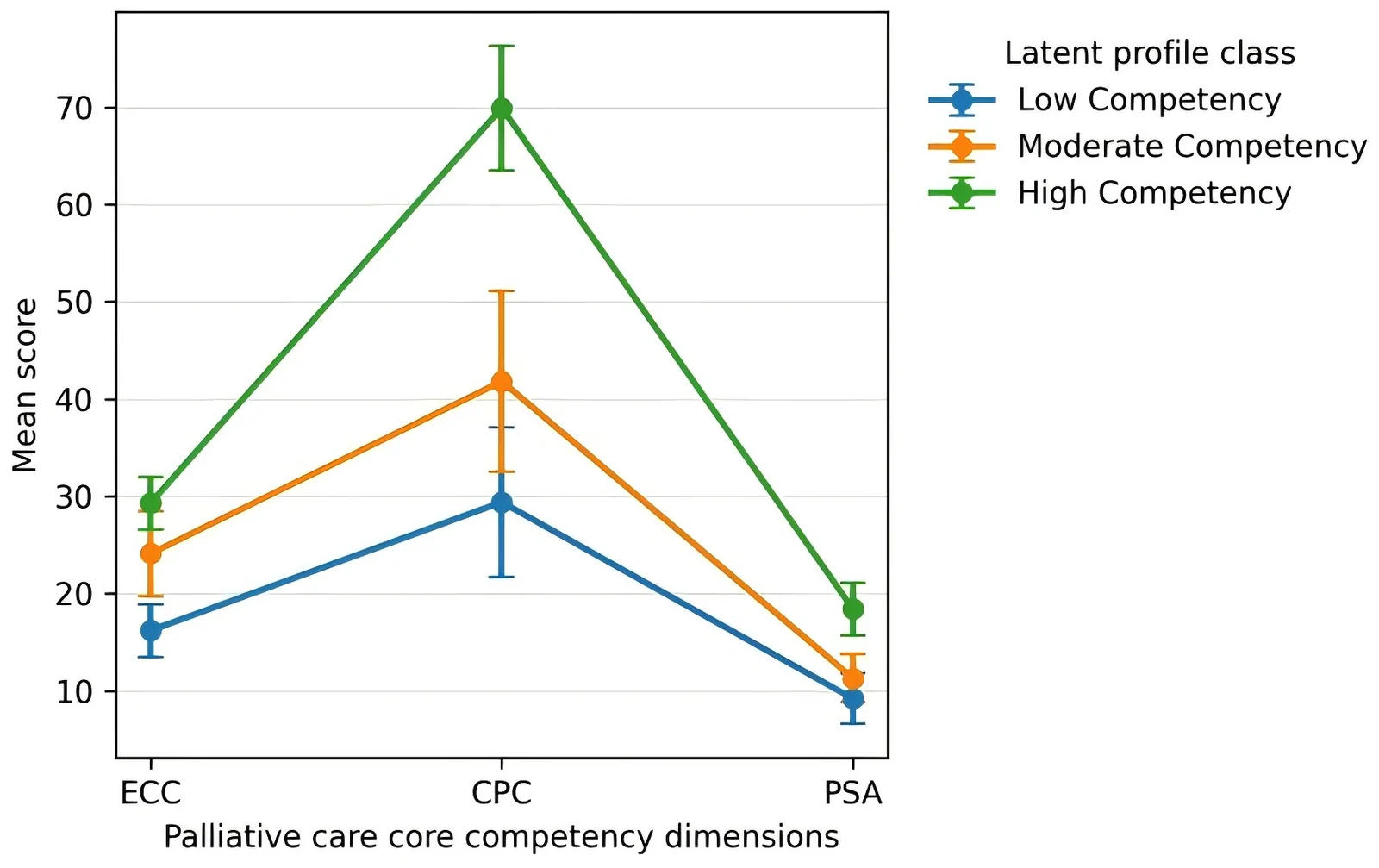
Mean scores across dimensions in the latent profile analysis. Three distinct profiles were identified: Low Competency, Moderate Competency, and High Competency. Error bars represent standard deviations. Abbreviations: ECC, Ethical Care Competency; CPC, Clinical Practice Competency; PSA, Psychological Self-Adjustment Ability.

Three profiles emerged: low-competency (27%, *n* = 65; scores: ECC 16.2 ± 2.7, CPC 29.4 ± 7.7, PSA 9.2 ± 2.6), moderate-competency (45%, *n* = 108; ECC 24.1 ± 4.4, CPC 41.8 ± 9.3, PSA 11.3 ± 2.5), and high-competency (28%, *n* = 68; ECC 29.3 ± 2.7, CPC 69.9 ± 6.4, PSA 18.4 ± 2.7) (Fig. 1). Intergroup differences were significant in both the total score of core competency and all dimensions (all *P* < 0.001). High-competency scores exceeded low-competency by 1.8-fold (ECC), 2.4-fold (CPC), and 2.0-fold (PSA). These differences indicate not only statistically significant separation among profiles but also clinically meaningful heterogeneity in ethical care, clinical practice, and psychological self-adjustment capacities. Differences across profiles were further examined using univariate analyses. Several demographic and occupational variables differed across latent profiles, including sex, hospital grade, palliative care training, recent bereavement, and work interest (all *P* < 0.050; Table S1).

### External validity verification

**Figure 2:**
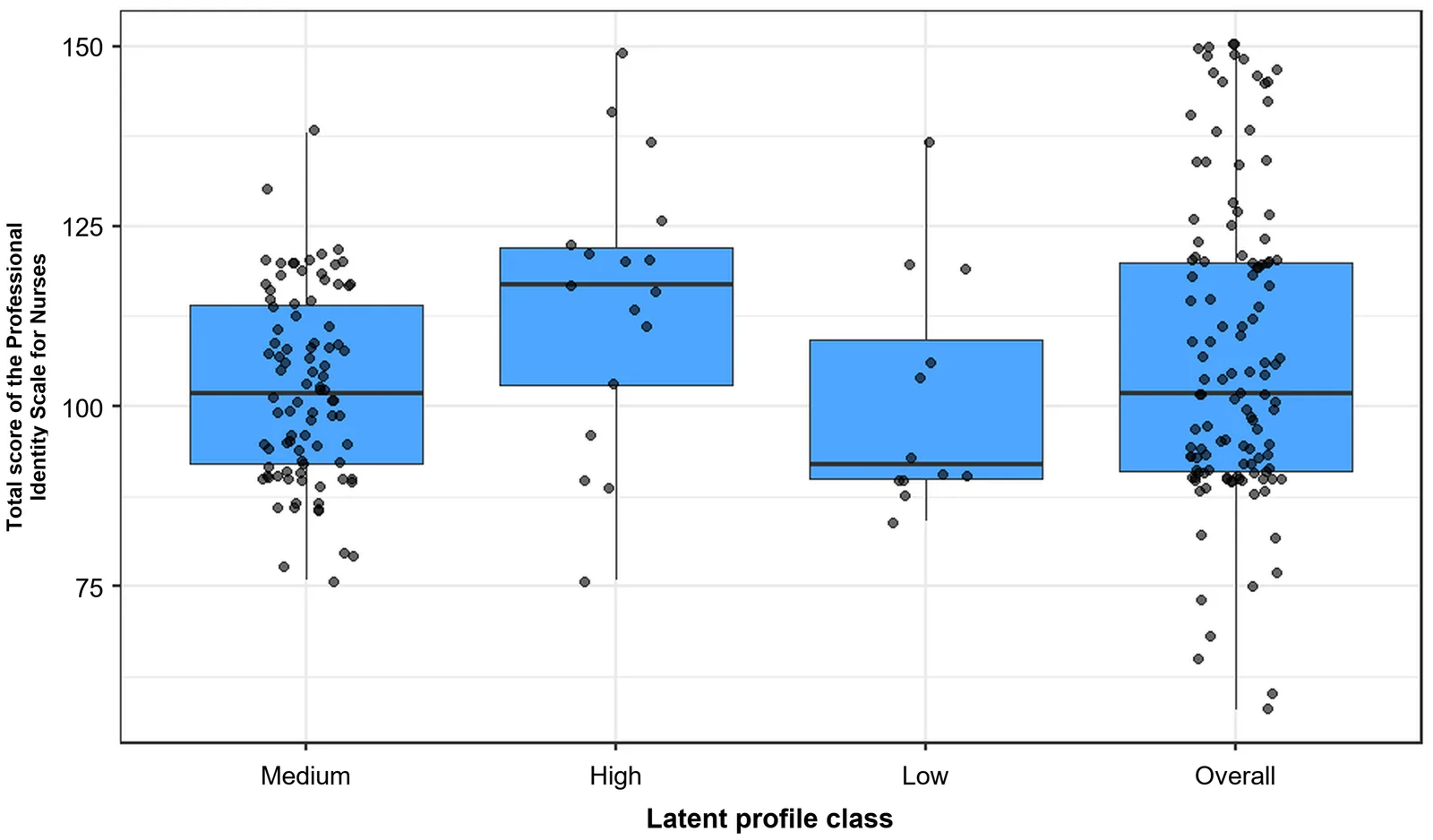
Differences in nurses’ total scores of professional identity among the three latent profiles. The high competency group demonstrated significantly higher professional identity scores compared to the moderate and low competency groups.

Professional identity was used as an external criterion to examine whether the identified latent profiles reflected meaningful differences beyond the profile indicators. There were significant differences in the total scores of professional identities among the three groups (F (2,238) = 24.11, *P* < 0.001, *η*^2^ = 0.17), with a large effect size. The high-competency group had the highest score (116.91 ± 22.22), followed by the moderate-competency group (104.59 ± 4.60), and the low-ability group had the lowest score (96.68 ± 14.26). *Post hoc* comparisons showed that the differences between each group were significant, supporting the external validity and practical meaningfulness of the latent profiles (Fig. 2).

### Influencing factors for latent profiles

Univariate analyses showed significant differences across profiles in sex, hospital grade, palliative training, recent bereavement, and work interest (all *P* < 0.050; Table S1). Multivariate logistic regression, using the moderate-competency group as the reference category, was conducted to examine factors associated with membership in the low- and high-competency profiles. After adjustment for demographic and occupational variables included in the model, nurses who received palliative care training were more likely to be classified into the high-competency group than into the moderate-competency group (*B* = 1.166, *OR* = 3.210, 95% CI [1.298–7.940]; *P* = 0.012). Higher average monthly night-shift frequency was associated with lower odds of being classified into the low-competency group rather than the moderate-competency group (*B* = −0.120, *OR* = 0.887, 95% CI [0.788–0.998]; *P* = 0.046). Because the confidence interval was close to 1.000, this association should be interpreted as modest and exploratory. Absence of religious affiliation was also associated with lower odds of low-competency group membership compared with having a religious affiliation (*B* = −2.369, *OR* = 0.094, 95% CI [0.010–0.893]; *P* = 0.040). However, this finding should be interpreted cautiously because the number of participants with religious affiliation was very small. Owing to the small sample sizes in several categorical variables, such as gender, education level, religious affiliation, and some marital-status categories, several OR estimates showed substantial uncertainty and should be interpreted as exploratory rather than confirmatory. Validation through larger samples is needed (Table 3). Detailed effect sizes (*η*^2^ with 95% CI), *post-hoc* pairwise comparisons (Cohen’s d), and multiple comparison corrections (Bonferroni, Holm, FDR) are shown in Table S2.

**Table 3:** Multivariate logistic regression analysis of factors associated with latent profile membership (*N* = 241).

Variable	Low competency group (*vs.* moderate group)		High competency group (*vs.* moderate group)	
	*OR (95% CI)*	*P*	*OR (95% CI)*	*P*
Average monthly night shifts	0.89 (0.79–0.99)	**0.046**	0.95 (0.84–1.07)	0.395
Received palliative care training (ref: No)	0.71 (0.32–1.61)	0.417	3.21 (1.30–7.94)	**0.012**
Religious affiliation (ref: Present)	0.09 (0.01–0.89)	**0.040**	0.54 (0.03–8.48)	0.661

**Notes.**

1. The Moderate Competency Group served as the reference category.

2. Data are presented as odds ratios (OR) with 95% confidence intervals (CI).

3. Variables with sparse categories were considered in the initial model but are not presented because they produced unstable or non-estimable estimates. Marital status was excluded from the final model because the divorced/widowed category contained only two participants (*n* = 2), resulting in unstable parameter estimates. Therefore, only the core predictors retained for interpretation are shown in this table.

4. Significant predictors (*P* < 0.05) are highlighted in bold.

5. Abbreviations: OR, odds ratio; CI, confidence interval; ref., reference category.

### Robustness analysis

Non-parametric bootstrap analysis with 1,000 resamples was conducted to examine the stability of the multivariate logistic regression findings. The association between palliative care training and membership in the high-competency group remained directionally consistent with the main regression model. The estimates for average monthly night-shift frequency and religious affiliation should be interpreted cautiously because they were close to the threshold of statistical significance and were affected by sparse or unevenly distributed categories. After sparse categories were merged, the main conclusion regarding the positive association between palliative care training and high-competency profile membership remained generally consistent. The total scale demonstrated excellent reliability (α = 0.979, 95% CI [0.977–0.982]; ωt = 0.981). Subdomain reliabilities were: ECC (α = 0.942, ωt = 0.953), CPC (α = 0.978, ωt = 0.980), and PSA (α = 0.948, ωt = 0.960). The Omega Hierarchical (ωh = 0.618) supported the multidimensional structure, suggesting that subdomain scores should be interpreted alongside the total score.

## Discussion

This study identified three distinct latent profiles of palliative care core competency among oncology nurses from five hospitals in Shenzhen: low-, moderate-, and high-competency groups. These profiles represent not only graduated differences in overall ability but also a structural imbalance, characterized by comparatively strong ethical care competency, moderate clinical practice competency, and markedly weaker psychological self-adjustment. This pattern underscores the multidimensional nature of palliative care competency and suggests that psychological self-adjustment may be a relatively weak domain requiring greater attention in future competency-development programs, including emotional support, stress-coping resources, and resilience-oriented training. Our findings extend existing knowledge by applying a person-centered analytic approach to uncover heterogeneity that traditional variable-centered methods tend to obscure.

Compared with the three-profile classification identified in the latent profile analysis of palliative care competency among 342 ICU nurses by Guan et al. (2025), the average core palliative care competency score among ICU nurses was 58.96 ± 21.56. Three distinct palliative care core competency subgroups were identified: the low-competency group (31.2%), the moderate-competency group (47.2%), and the high-competency group (21.6%). This study aligns with these findings in having the highest proportion in the moderate competency group, although our proportion remains slightly lower than that reported in that study. The higher proportion of nurses in the high-competency group may be due to differences in clinical setting, training exposure and regional resources. Since this study uses cross-sectional design and regional sample, these explanations should be considered as possible contexts rather than as confirmed mechanisms (Li et al., 2025).

The shared decision-making model emphasizes that when patients transition from curative treatment to palliative care; nurses functioning as communication facilitators and ethical care providers may play an important role in supporting end-of-life decision-making and maintaining patient-centered care (Kuosmanen et al., 2021; Washington et al., 2022). The low-competency group in this study scored only 16.2 points in ethical care, suggesting that nurses in this profile may require further support in ethical communication, patient preference clarification, and multidisciplinary decision-making in complex palliative care situations.

Symptom management theory further indicates that symptoms in terminal cancer patients often manifest as symptom clusters, requiring nurses to conduct comprehensive assessment and individualized management (Dodd et al., 2001). The clinical practice score of the low-competency group in this study (29.4 ± 7.7) was less than half that of the high-competency group, indicating a substantial gap in clinical practice competency between profiles. This difference may be related to variation in clinical exposure and opportunities to manage complex symptoms, but this explanation should be regarded as contextual rather than causal because of the cross-sectional design. In the multivariate model, higher average monthly nightshift frequency was slightly associated with a lower probability of membership in the low-competency group. However, because the estimate was close to the null value, this finding should be regarded as exploratory rather than evidence that night-shift work improves competency.

Religious affiliation also showed an association with latent profile membership in the multivariate model. However, this result should be interpreted with particular caution because only seven participants reported religious affiliation. Although relevant studies suggest that conflicts between religious “redemption” narratives and the clinical reality of death may be related to existential anxiety among nurses (Hossain, Gabbay & Fins, 2025), the present finding should not be interpreted as evidence of a direct effect of religious belief on ethical decision-making or palliative care competency. Given the small number of participants with religious affiliation, this association may reflect unmeasured cultural, spiritual, or contextual factors and requires further validation through subsequent mixed-methods research (Melender et al., 2020).

Using professional identity as an external criterion, the between-profile effect size reached *η*^2^ = 0.17 (large effect), indicating that palliative care core competency profiles and professional identity formation were meaningfully related. This suggests that enhancing professional identity may serve as one potential direction for supporting competency development. Although the causal direction between professional identity and competency cannot be determined in this cross-sectional study.

This study introduces LPA to dissect palliative care competencies among Chinese oncology nurses, advancing beyond aggregate metrics to reveal nuanced subgroup dynamics. The integration of shared decision-making and symptom management theories provides a conceptual framework for interpreting the multidimensional competency patterns identified in this study. Robust model validation through multi-index fitting, bootstrap resampling, and external criteria strengthens confidence in the profile solution. However, the findings should be interpreted in light of the study design and sample characteristics, and they should be used to inform, rather than definitively establish, targeted competency-development strategies. However, our study has the following limitations. The cross-sectional design precludes causal inferences regarding the relationships between training, demographic factors, and competency profiles (Levin, 2006). While systematic training was associated with higher odds of high-competency membership, we cannot determine whether training causes competency improvement or whether nurses with higher baseline competency are more likely to pursue training. Longitudinal or randomized controlled trials would be required to establish causality and temporal sequence. The sample, drawn from five Shenzhen hospitals, may limit generalization to rural or non-specialized contexts. Self-reported data introduce potential response biases, including social desirability, and small subgroup sizes (*e.g.*, for gender/education) contributed to unstable estimates in logistic models. Objective measures (*e.g.*, OSCE simulations) and larger, multi-center cohorts could mitigate these issues.

## Conclusions

Our cross-sectional survey analysis of 241 nurses from five hospitals in Shenzhen revealed a tripartite “low-moderate-high” profile distribution in palliative care core competency. Systematic training was associated with higher odds of high-competency membership (OR = 3.21), suggesting it may be a potentially modifiable factor for competency enhancement. Average monthly night-shift frequency and religious affiliation showed exploratory associations with profile membership.However, these findings should be interpreted cautiously because the estimates were close to the null value or affected by sparse subgroup categories. Future longitudinal and interventional studies are needed to test whether tiered training programs and psychological support interventions can effectively enhance palliative care competency, particularly in the psychological self-adjustment domain. In addition, such studies should clarify the temporal relationships between training, professional development, and competency outcomes.

## Supplemental Information

10.7717/peerj.21645/supp-1 Supplemental Information 1 Abbreviation List

10.7717/peerj.21645/supp-2 Supplemental Information 2 Univariate Analysis of General Characteristics and Latent Profiles of Hospice Care Core Competency

10.7717/peerj.21645/supp-3 Supplemental Information 3 Comparison of Core Competencies and Professional Identity across Three Latent Profiles (*N* = 241)

10.7717/peerj.21645/supp-4 Supplemental Information 4 STROBE Checklist

10.7717/peerj.21645/supp-5 Supplemental Information 5 Raw data in English

10.7717/peerj.21645/supp-6 Supplemental Information 6 Raw data in Chinese

10.7717/peerj.21645/supp-7 Supplemental Information 7 Data annotation

10.7717/peerj.21645/supp-8 Supplemental Information 8 Chinese & English language codebook

## Additional Information and Declarations

### Data Availability

The following information was supplied regarding data availability: The data is available in the Supplemental Files.

## References

[ref-1] Barry MJ, Edgman-Levitan S (2012). Shared decision making–pinnacle of patient-centered care. The New England Journal of Medicine.

[ref-2] Bray F, Laversanne M, Sung H, Ferlay J, Siegel RL, Soerjomataram I, Jemal A (2024). Global cancer statistics 2022: GLOBOCAN estimates of incidence and mortality worldwide for 36 cancers in 185 countries. CA: A Cancer Journal for Clinicians.

[ref-3] Dodd M, Janson S, Facione N, Faucett J, Froelicher ES, Humphreys J, Lee K, Miaskowski C, Puntillo K, Rankin S, Taylor D (2001). Advancing the science of symptom management. Journal of Advanced Nursing.

[ref-4] Guan Q, Zhu X, Xue Z, Peng M (2025). Core competency in palliative care among intensive care unit nurses: a latent profile analysis. Nursing in Critical Care.

[ref-5] Han G (2021). Study on the current status of core competencies of hospice nurses and the development of a training program. Master’s thesis.

[ref-6] Han B, Zheng R, Zeng H, Wang S, Sun K, Chen R, Li L, Wei W, He J (2024). Cancer incidence and mortality in China, 2022. Journal of the National Cancer Center.

[ref-7] Hart NH, Crawford-Williams F, Crichton M, Yee J, Smith TJ, Koczwara B, Fitch MI, Crawford GB, Mukhopadhyay S, Mahony J, Cheah C, Townsend J, Cook O, Agar MR, Chan RJ (2022). Unmet supportive care needs of people with advanced cancer and their caregivers: a systematic scoping review. Critical Reviews in Oncology/ Hematology.

[ref-8] Hossain F, Gabbay E, Fins JJ (2025). Clinical ethics and the observant jewish and muslim patient: shared theocentric perspectives in practice. Cambridge Quarterly of Healthcare Ethics: CQ: The International Journal of Healthcare Ethics Committees.

[ref-9] Kuosmanen L, Hupli M, Ahtiluoto S, Haavisto E (2021). Patient participation in shared decision-making in palliative care–an integrative review. Journal of Clinical Nursing.

[ref-10] Levin KA (2006). Study design III: cross-sectional studies. Evidence-Based Dentistry.

[ref-11] Li J, Arber A, Chen X, Chen Y, Sun C, Wu J, Chen X (2025). Latent class analysis of death coping ability among palliative care nurses and its association with their emotional labor. BMC Palliative Care.

[ref-12] Li W-Y, Fang Y, Liang Y-Q, Zhu S-Q, Yuan L, Xu Q, Li Y, Chen Y-L, Sun C-X, Zhi X-X, Li X-Y, Zhou R, Du M (2023). Building bridges of excellence: a comprehensive competence framework for nurses in hospice and palliative care-a mixed method study. BMC Palliative Care.

[ref-13] Liu L (2009). Research on nurses’ professional identity and its relationship with job stress and burnout. Master’s thesis.

[ref-14] MacCallum RC, Widaman KF, Preacher KJ, Hong S (2001). Sample size in factor analysis: the role of model error. Multivariate Behavioral Research.

[ref-15] Melender H-L, Hökkä M, Saarto T, Lehto JT (2020). The required competencies of physicians within palliative care from the perspectives of multi-professional expert groups: a qualitative study. BMC Palliative Care.

[ref-16] Nouhi E, Faramarzpour V, Shahrbabaki PM (2021). Iranian nurses’ educational needs and competence in palliative cancer care. International Journal of Palliative Nursing.

[ref-17] Paatela S, Pohjamies N, Kanste O, Happa T, Oikarainen A, Kääriäinen M, Mikkonen K (2024). Registered nurses’ cultural orientation competence for culturally and linguistically diverse nurses in the hospital setting: a cross-sectional study. Journal of Advanced Nursing.

[ref-18] Sharratt P, Zacharias A, Nwosu AC, Gadoud A (2024). Hospital-initiated palliative care interventions for adults with frailty: findings from a systematic review and narrative synthesis. Age and Ageing.

[ref-19] Silva LAGP da, Lopes VJ, Mercês NNA das (2021). Symptom management theory applied to nursing care: scoping review. Revista Brasileira De Enfermagem.

[ref-20] Tiantian W, Jie C, Nanxiao REN, Yunrong LI, Liuliu Z, Bing WU, Yun Z (2025). Current status and latent profile analysis of nurses’ caring behaviors in hospice care. Chinese Journal of Nursing.

[ref-21] Von Elm E, Altman DG, Egger M, Pocock SJ, Gøtzsche PC, Vandenbroucke JP (2007). Strengthening the reporting of observational studies in epidemiology (STROBE) statement: guidelines for reporting observational studies. BMJ: British Medical Journal.

[ref-22] Washington KT, Demiris G, White P, Mathis HC, Forsythe JE, Parker Oliver D (2022). A goal-directed model of collaborative decision making in hospice and palliative care. Journal of Palliative Care.

[ref-23] Woo OKL, Lee AM (2023). Case report: therapeutic potential of flourishing-life-of-wish virtual reality therapy on relaxation (FLOW-VRT-Relaxation)-a novel personalized relaxation in palliative care. Frontiers in Digital Health.

[ref-24] Zhang J, Fang H, Sun Y, Wang W, Yuan Y, Zheng K (2024). Meta-analysis of palliative care on end-stage quality of life in cancer patients. Alternative Therapies in Health and Medicine.

[ref-25] Zhao J, Wang Y, Xiao B, Ye F, Chen J, Huang Y, Li T, Chen X, Ma H, Zhang Q, Zou Z (2024). Behaviors and influencing factors of Chinese oncology nurses towards hospice care: a cross-sectional study based on social cognitive theory in 2022. BMC Palliative Care.

