# Supplementary material for: Heterogeneity and associated factors of palliative care core competency among oncology nurses: a latent profile analysis
Source: PeerJ. 2026 Aug 26;14:e21645. doi: 10.7717/peerj.21645 (PMC13526326; doi:10.7717/peerj.21645)
Supplement: Supplementary file 1 [file peerj-14-21645-s001.docx]

**Abbreviation List**

| **Abbreviation** | **Full Name** |
| --- | --- |
| ECC | Ethical Care Competency |
| CPC | Clinical Practice Competency |
| PSA | Psychological Self-Adjustment Ability |
| LPA | Latent Profile Analysis |
| OR | Odds Ratio |
| CI | Confidence Interval |
| BLRT | Bootstrap Likelihood Ratio Test |
| LMR | Lo-Mendell-Rubin Test |
| AIC | Akaike Information Criterion |
| BIC | Bayesian Information Criterion |
| aBIC | Adjusted Bayesian Information Criterion |
| OSCE | Objective Structured Clinical Examination |
