# Supplementary material for: Heterogeneity and associated factors of palliative care core competency among oncology nurses: a latent profile analysis
Source: PeerJ. 2026 Aug 26;14:e21645. doi: 10.7717/peerj.21645 (PMC13526326; doi:10.7717/peerj.21645)
Supplement: Supplementary file 2 [file peerj-14-21645-s002.docx]

**Supplementary Table S1. Univariate Analysis of General Characteristics and Latent Profiles of Hospice Care Core Competency**

| Variable | Total cases (N=241) | Low level (N=65) | Moderate level (N=108) | High level (N=68) | X^2^/F | *P* |
| --- | --- | --- | --- | --- | --- | --- |
| Age | 32.98±6.70 | 31.95±6.41 | 33.59±6.39 | 32.97±7.39 | 1.217 | 0.298 |
| Gender |  |  |  |  | 6.306 | 0.043 |
| Male | 8（3.3） | 5（7.7） | 3（92.3） | 0（0.0） |  |  |
| Female | 233（96.7） | 60（92.3） | 105（97.2） | 68（100.0） |  |  |
| Education level |  |  |  |  | 6.541 | 0.365 |
| Associate degree or below | 22（9.1） | 9（13.8） | 8（7.4） | 5（7.4） |  |  |
| Bachelor's degree | 207（85.9） | 55（84.6） | 94（87.0） | 58（85.3） |  |  |
| Master's degree | 11（4.6） | 1（1.5） | 6（5.6） | 4（5.9） |  |  |
| Doctoral degree | 1（0.4） | 0（0.0） | 0（0.0） | 1（1.5） |  |  |
| Marital status |  |  |  |  | 3.750 | 0.441 |
| Unmarried | 83（34.4） | 26（40.0） | 31（28.7） | 26（38.2） |  |  |
| Married | 156（64.7） | 39（60.0） | 76（70.4） | 41（60.3） |  |  |
| Divorced or widowed | 2（0.8） | 0（0.0） | 1（0.9） | 1（1.5） |  |  |
| Ethnicity |  |  |  |  | 1.045 | 0.593 |
| Han Chinese | 223（92.5） | 59（90.8） | 102（94.4） | 62（91.2） |  |  |
| Other | 18（7.5） | 6（9.2） | 6（5.6） | 6（8.8） |  |  |
| Religious affiliation |  |  |  |  | 1.099 | 0.577 |
| No | 234（97.1） | 62（95.4） | 106（98.1） | 66（97.1） |  |  |
| Yes | 7（2.9） | 3（4.6） | 2（1.9） | 2（2.9） |  |  |
| Hospital classification |  |  |  |  | 17.080 | 0.009 |
| Tertiary hospital | 182（75.5） | 40（61.5） | 81（75.0） | 61（89.7） |  |  |
| Secondary hospital | 22（9.1） | 12（18.5） | 9（8.3） | 1（1.5） |  |  |
| Primary hospital | 31（12.9） | 11（16.9） | 15（13.9） | 5（7.4） |  |  |
| Senior care institution | 6（2.5） | 2（3.1） | 3（2.8） | 1（1.5） |  |  |
| Department |  |  |  |  | 11.297 | 0.185 |
| Hospice care ward | 24（10.0） | 5（7.7） | 14（13.0） | 5（7.4） |  |  |
| Oncology department | 141（58.5） | 34（52.3） | 59（54.6） | 48（70.6） |  |  |
| Pain management department | 0（0.0） | 0（0.0） | 0（0.0） | 0（0.0） |  |  |
| Integrated Chinese and Western medicine department | 6（2.5） | 3（4.6） | 1（0.9） | 2（2.9） |  |  |
| Geriatrics department | 18（7.5） | 4（6.2） | 10（9.3） | 4（5.9） |  |  |
| Other | 52（21.6） | 19（29.2） | 24（22.2） | 9（13.2） |  |  |
| Average monthly night shifts | 4.69±3.33 | 4.35±3.04 | 4.96±3.60 | 4.57±3.15 | 0.733 | 0.481 |
| Years of employment | 11.23±7.09 | 10.12±7.07 | 12.23±7.32 | 10.71±6.62 | 2.073 | 0.128 |
| Duration in hospice care | 11.09±30.33 | 5.72±11.55 | 9.94±15.58 | 18.03±51.98 | 2.920 | 0.056 |
| Average monthly income |  |  |  |  | 3.509 | 0.477 |
| Below 3000 RMB | 3（1.2） | 1（1.5） | 0（0.0） | 2（2.9） |  |  |
| 3000-5000 RMB | 28（1.6） | 6（9.2） | 14（13.0） | 8（11.8） |  |  |
| Above 5000 RMB | 210（87.1） | 58（89.2） | 94（87.0） | 58（85.3） |  |  |
| Whether monthly income is  within expected range |  |  |  |  | 3.082 | 0.214 |
| Yes | 140（58.1） | 38（58.5） | 57（52.8） | 45（66.2） |  |  |
| No | 101（41.9） | 27（41.5） | 51（47.2） | 23（33.8） |  |  |
| Professional title |  |  |  |  | 2.745 | 0.949 |
| Staff Nurse | 31（12.9） | 10（15.4） | 12（11.1） | 9（13.2） |  |  |
| Nurse Practitioner | 82（34.0） | 22（33.8） | 35（32.4） | 25（36.8） |  |  |
| Charge Nurse | 112（46.5） | 29（44.6） | 54（50.0） | 29（42.6） |  |  |
| Associate Director Nurse | 13（5.4） | 4（6.2） | 5（4.6） | 4（5.9） |  |  |
| Director Nurse | 3（1.2） | 0（0.0） | 2（1.9） | 1（1.5） |  |  |
| Whether serving as clinical instructor |  |  |  |  | 2.001 | 0.368 |
| Yes | 100（41.5） | 26（40.0） | 41（38.0） | 33（48.5） |  |  |
| No | 141（58.5） | 39（60.0） | 67（62.0） | 35（51.5） |  |  |
| Have you received hospice care  training? |  |  |  |  | 10.144 | 0.006 |
| Yes | 154（63.9） | 36（55.4） | 64（59.3） | 54（79.4） |  |  |
| No | 87（36.1） | 29（44.6） | 44（40.7） | 14（20.6） |  |  |
| Have you experienced the loss of a loved one in the past year? |  |  |  |  | 8.995 | 0.011 |
| Yes | 47（19.5） | 20（30.8） | 20（18.5） | 7（10.3） |  |  |
| No | 194（80.5） | 45（69.2） | 88（81.5） | 61（89.7） |  |  |
| Have you cared for  terminally ill patients? |  |  |  |  | 1.644 | 0.440 |
| Yes | 209（86.7） | 55（84.6） | 92（85.2） | 62（91.2） |  |  |
| No | 32（13.3） | 10（15.4） | 16（14.8） | 6（8.8） |  |  |
| How interested are you in  hospice care work? |  |  |  |  | 13.240 | 0.039 |
| Very interested | 50（20.7） | 13（20.0） | 16（14.8） | 21（30.9） |  |  |
| Interested | 121（50.2） | 31（47.7） | 53（49.1） | 37（54.4） |  |  |
| Neutral | 67（27.8） | 20（30.8） | 38（35.2） | 9（13.2） |  |  |
| Not interested | 3（1.2） | 1（1.5） | 1（0.9） | 1（1.5） |  |  |
| Have you considered changing careers? |  |  |  |  | 1.122 | 0.571 |
| Yes | 61（25.3） | 19（29.2） | 24（22.2） | 18（26.5） |  |  |
| No | 180（74.7） | 46（70.8） | 84（77.8） | 50（73.5） |  |  |

**Note:**

1. Data are presented as mean ± standard deviation for continuous variables and number (percentage) for categorical variables.
2. The three latent profiles (Low, Moderate, and High competency) were identified through latent profile analysis.
3. Group comparisons were performed using one-way ANOVA for continuous variables and chi-square tests for categorical variables.
4. Significant P-values (P < 0.05) are highlighted in bold.
5. Variables such as hospital classification, receipt of hospice care training, experience of recent bereavement, and interest in hospice work showed significant differences across the competency profiles.
