# Supplementary material for: Heterogeneity and associated factors of palliative care core competency among oncology nurses: a latent profile analysis
Source: PeerJ. 2026 Aug 26;14:e21645. doi: 10.7717/peerj.21645 (PMC13526326; doi:10.7717/peerj.21645)
Supplement: Supplementary file 3 [file peerj-14-21645-s003.docx]

**Supplementary Table S2-1. Comparison of Core Competencies and Professional Identity across Three Latent Profiles (N = 241)**

| **Variable** | **Profile 1 (n = 65)** | **Profile 2 (n = 108)** | **Profile 3 (n = 68)** | **F(2, 238)** | **p** | **η² [95% CI]** |
| --- | --- | --- | --- | --- | --- | --- |
| Psychological Care (ECC) | 16.2 ± 2.7 | 24.1 ± 4.4 | 29.3 ± 2.7 | 227.60 | < .001** | 0.66 [0.58, 0.74] |
| Communication (CPC) | 29.4 ± 7.7 | 41.8 ± 9.3 | 69.9 ± 6.4 | 441.70 | < .001** | 0.79 [0.73, 0.84] |
| Psychological Self-adjustment (PSA) | 9.2 ± 2.6 | 11.3 ± 2.5 | 18.4 ± 2.7 | 240.80 | < .001** | 0.67 [0.59, 0.75] |
| Professional Identity | 96.7 ± 14.3 | 104.6 ± 14.6 | 116.9 ± 22.2 | 24.11 | < .001** | 0.17 [0.02, 0.32] |

*Note. Values are Mean ± SD. η² = eta-squared. ANOVA = analysis of variance. **p < .001 after Bonferroni correction (α' = .00417). All effect sizes are large (η² > 0.14) according to Cohen (1988).*

**Supplementary Table S2-2. Post-hoc Pairwise Comparisons with Effect Sizes**

| **Comparison** | **Variable** | **Mean Diff** | **Cohen's d** | **95% CI** | **Adj. p** |
| --- | --- | --- | --- | --- | --- |
| Profile 1 vs 2 | Psychological Care (ECC) | -7.90 | -2.06 | [-2.42, -1.70] | < .001*** |
|  | Communication (CPC) | -12.40 | -1.43 | [-1.74, -1.12] | < .001*** |
|  | Psychological Self-adjustment (PSA) | -2.10 | -0.83 | [-1.11, -0.55] | < .001*** |
|  | Professional Identity | -7.91 | -0.55 | [-0.86, -0.23] | .008** |
| Profile 1 vs 3 | Psychological Care (ECC) | -13.10 | -4.85 | [-5.57, -4.13] | < .001*** |
|  | Communication (CPC) | -40.50 | -5.73 | [-6.45, -5.01] | < .001*** |
|  | Psychological Self-adjustment (PSA) | -9.20 | -3.47 | [-4.05, -2.89] | < .001*** |
|  | Professional Identity | -20.23 | -1.08 | [-1.44, -0.72] | < .001*** |
| Profile 2 vs 3 | Psychological Care (ECC) | -5.20 | -1.35 | [-1.68, -1.02] | < .001*** |
|  | Communication (CPC) | -28.10 | -3.38 | [-3.86, -2.90] | < .001*** |
|  | Psychological Self-adjustment (PSA) | -7.10 | -2.74 | [-3.16, -2.32] | < .001*** |
|  | Professional Identity | -12.32 | -0.69 | [-0.99, -0.38] | < .001*** |

*Note. Mean Diff = Mean difference (Group 1 - Group 2). Adj. p = Bonferroni-corrected p-value. Cohen's d interpretation: small ≥ 0.20, medium ≥ 0.50, large ≥ 0.80. ***p < .001, **p < .01 after Bonferroni correction (α' = .00417).*

**Supplementary Table S2-3. Multiple Comparison Correction Results for 12 Independent Pairwise Comparisons**

| **Variable** | **Comparison** | **Raw p** | **Bonferroni p** | **Holm p** | **FDR p** |
| --- | --- | --- | --- | --- | --- |
| Psychological Care (ECC) | Profile 1 vs 2 | < .001 | < .001*** | < .001 | < .001 |
|  | Profile 1 vs 3 | < .001 | < .001*** | < .001 | < .001 |
|  | Profile 2 vs 3 | < .001 | < .001*** | < .001 | < .001 |
| Communication (CPC) | Profile 1 vs 2 | < .001 | < .001*** | < .001 | < .001 |
|  | Profile 1 vs 3 | < .001 | < .001*** | < .001 | < .001 |
|  | Profile 2 vs 3 | < .001 | < .001*** | < .001 | < .001 |
| Psychological Self-adjustment (PSA) | Profile 1 vs 2 | < .001 | < .001*** | < .001 | < .001 |
|  | Profile 1 vs 3 | < .001 | < .001*** | < .001 | < .001 |
|  | Profile 2 vs 3 | < .001 | < .001*** | < .001 | < .001 |
| Professional Identity | Profile 1 vs 2 | < .001 | .008** | < .001 | < .001 |
|  | Profile 1 vs 3 | < .001 | < .001*** | < .001 | < .001 |
|  | Profile 2 vs 3 | < .001 | < .001*** | < .001 | < .001 |

*Note. N = 241. Total number of comparisons = 12 (4 variables × 3 pairwise comparisons). Bonferroni threshold: α' = .00417 (.05/12); Holm: step-down procedure; FDR: Benjamini-Hochberg false discovery rate. ***p < .001, **p < .01 after Bonferroni correction. Key Finding: All 12 comparisons remained statistically significant after conservative Bonferroni correction, indicating robust differences across latent profiles.*
