# Supplementary material for: Heterogeneity and associated factors of palliative care core competency among oncology nurses: a latent profile analysis
Source: PeerJ. 2026 Aug 26;14:e21645. doi: 10.7717/peerj.21645 (PMC13526326; doi:10.7717/peerj.21645)
Supplement: Supplementary file 5 [file peerj-14-21645-s008.docx]

一般资料表中英文对照/General Information Form (Chinese-English Comparison)

| 中文问题 | English translation |
| --- | --- |
| 1. 您的年龄: | 1. Your Age: |
| 2. 您的性别: | 2. Your Gender: |
| 3. 您的文化程度: | 3. Your Education Level: |
| 4. 您的婚姻状况: | 4. Your Marital Status: |
| 5. 您的民族为: | 5. Your Ethnicity: |
| 6. 您的宗教信仰为: | 6. Your Religious Belief: |
| 7. 您所在工作医院性质是 | 7. The Nature of the Hospital You Work In: |
| 8. 您所在工作科室为: | 8. The Department You Work In: |
| 9. 平均月夜班值班数:___ | 9. Average Number of Night Shifts per Month: ___ |
| 10. 您的工作年限（总）:___安宁疗护工作时间（月）:___ | 10. Total Working Experience (Years): ___Working Experience in Hospice Care (Months): ___ |
| 11. 您的平均月收入为: | 11. Your Average Monthly Income: |
| 12. 您的月收入是否在您期望的范围内: | 12. Is Your Monthly Income Within Your Expected Range? |
| 13. 您的职称为: | 13. Your Professional Title: |
| 14. 您是通过何种途径学习安宁疗护知识的呢?(讲座) □ (会议) □ (专业杂志) □ (教科书) □(教育/培训) □ (进修) □ (电视/网络) □ (其他) □ | 14. What Channels Have You Used to Learn Hospice Care Knowledge?(Lectures) □ (Conferences) □ (Professional Journals) □ (Textbooks) □(Education/Training) □ (Advanced Studies) □ (TV/Internet) □ (Others) □ |
| 15. 您是否担任带教: | 15. Do You Take on Teaching Responsibilities for Trainees? |
| 16. 您更倾向于通过何种方向学习安宁疗护知识?(讲座) □ (会议) □ (专业杂志) □ (教科书) □(教育/培训) □ (进修) □ (电视/网络) □ (其他) □ | 16. What Channels Do You Prefer for Learning Hospice Care Knowledge?(Lectures) □ (Conferences) □ (Professional Journals) □ (Textbooks) □(Education/Training) □ (Advanced Studies) □ (TV/Internet) □ (Others) □ |
| 17. 您是否接受过安宁疗护/临终关怀方面的培训? | 17. Have You Received Training in Hospice Care/Palliative Care? |
| 18. 您最近一年内是否经历过亲人(朋友)的离世? | 18. Have You Experienced the Passing of a Relative or Friend in the Past Year? |
| 19. 您是否护理过终末期患者? | 19. Have You Ever Cared for Terminally Ill Patients? |
| 20. 您对安宁疗护工作的兴趣: | 20. Your Interest in Hospice Care Work: |
| 21. 您是否考虑过转行（从事与安宁疗护无关的工作）: | 21. Have You Considered Changing to a Job Unrelated to Hospice Care? |

**护士安宁疗护核心能力问卷：**选用韩鸽鸽等人编制的《安宁疗护护士核心能力问卷》，该问卷包含伦理照护能力、临床实践能力、自我心理调适能力 3 个维度，共 32 个条目。本问卷为自评问卷，拟采用 Likert5级评分法，0 分代表“没有能力”，1 分代表“有一点能力”，2 分代表“有一些能力”，3 分代表“有足够能力”，4 分代表“很有能力”，总分0～128分，得分越高表示护士安宁疗护核心能力越强。该问卷的内容效度指数（S - CVI/Ave）为 0.911，Cronbach's α 系数为 0.980，折半信度为 0. 907，重测信度为 0. 959，具有良好的信效度。

**Nurse's Core Competence Questionnaire for Palliative Care:**

The Core Competence Questionnaire for Palliative Care Nurses, compiled by Han Gege et al., was adopted for this study. This questionnaire consists of 3 dimensions (ethical care competence, clinical practice competence, and self - psychological adjustment competence) with a total of 32 items.

It is a self - rated questionnaire scored on a 5 - point Likert scale, where:

0 point = No competence

1 point = Slight competence

2 points = Moderate competence

3 points = Adequate competence

4 points = Strong competence

The total score ranges from 0 to 128, with higher scores indicating stronger core palliative care competence of nurses. The questionnaire demonstrated good reliability and validity, with a Scale - Level Content Validity Index/Average (S - CVI/Ave) of 0.911, a Cronbach’s α coefficient of 0.980, a split - half reliability coefficient of 0.907, and a test - retest reliability coefficient of 0.959.

| **序号** | **中文条目** | **English translation** |
| --- | --- | --- |
| **维度1 伦理照护能力（Ethical Care Competency, ECC）——8条** | | |
| 1 | 平等对待患者及家属 | Treat patients and their family members equally |
| 2 | 维护患者及家属的尊严 | Safeguard the dignity of patients and their family members |
| 3 | 尊重患者及家属的习俗和宗教信仰 | Respect the customs and religious beliefs of patients and their family members |
| 4 | 尊重患者及家属知晓病情的意愿 | Respect the wishes of patients and their family members to be informed of the illness condition |
| 5 | 尊重患者及家属的治疗意愿 | Respect the treatment wishes of patients and their family members |
| 6 | 尊重并理解患者的主观感受（如疼痛、不舒适） | Respect and understand the subjective feelings of patients (such as pain and discomfort) |
| 7 | 为患者及家属提供安静、舒适、隐私的环境 | Provide a quiet, comfortable and private environment for patients and their family members |
| 8 | 保护患者及家属所述隐私内容 | Protect the privacy shared by patients and their family members |
| **维度2 临床实践能力（Clinical Practice Competency, CPC）——18条** | | |
| 9 | 能根据识别及评估的结果照护患者 | Be able to care for patients based on the results of identification and assessment |
| 10 | 能及时识别不同年龄患者的负性情绪 | Be able to timely identify the negative emotions of patients of different ages |
| 11 | 能根据患者的个人特质及时有效处理其心理问题 | Be able to timely and effectively address the psychological problems of patients according to their personal characteristics |
| 12 | 能及时识别不同年龄患者家属的负性情绪 | Be able to timely identify the negative emotions of family members of patients of different ages |
| 13 | 能根据家属的个人特质及时有效处理其心理问题 | Be able to timely and effectively address the psychological problems of family members according to their personal characteristics |
| 14 | 能帮助患者家属应对丧亲之痛 | Be able to help the family members of patients cope with grief over the loss of a loved one |
| 15 | 能积极寻求心理咨询师帮助解决患者及家属的复杂心理问题 | Be able to actively seek help from psychological consultants to solve complex psychological problems of patients and their family members |
| 16 | 能评估患者的社会支持状况 | Be able to assess the social support status of patients |
| 17 | 能为患者及家属提供准确、完整的信息支持 | Be able to provide accurate and complete information support for patients and their family members |
| 18 | 能评估患者的灵性需求 | Be able to assess the spiritual needs of patients |
| 19 | 能运用灵性照护方法满足患者的灵性需求 | Be able to use spiritual care methods to meet the spiritual needs of patients |
| 20 | 能评估患者家属的灵性需求 | Be able to assess the spiritual needs of patients' family members |
| 21 | 能运用灵性照护方法满足家属的灵性需求 | Be able to use spiritual care methods to meet the spiritual needs of family members |
| 22 | 能针对不同年龄的患者实施死亡教育 | Be able to carry out death education for patients of different ages |
| 23 | 能针对不同年龄的患者家属实施死亡教育 | Be able to carry out death education for family members of patients of different ages |
| 24 | 能积极寻求专业人员帮助解决患者及家属的复杂灵性问题 | Be able to actively seek help from professionals to solve complex spiritual problems of patients and their family members |
| 25 | 能与患者（家属）沟通安宁疗护的相关信息 | Be able to communicate relevant information about hospice care with patients (and their family members) |
| 26 | 能与病人、家属及多学科团队协商制定照护计划 | Be able to negotiate with patients, their family members and the multidisciplinary team to formulate care plans |
| **维度3 自我心理调适能力（Self-Psychological Adjustment, SPA）——6条** | | |
| 27 | 与患者及家属沟通时具有同理心 | Show empathy when communicating with patients and their family members |
| 28 | 面对病人及家属的消极心理反应能理性控制情绪 | Be able to rationally control emotions when facing the negative psychological reactions of patients and their family members |
| 29 | 能识别自己所面临的职业浩劫/能量耗竭等问题 | Be able to identify problems such as occupational burnout/energy exhaustion that one is facing |
| 30 | 能自我调节负性情绪对工作的影响 | Be able to self-regulate the impact of negative emotions on work |
| 31 | 能利用社会支持资源调节工作压力 | Be able to use social support resources to adjust work pressure |
| 32 | 能正确认识和评价自身角色，保持正常工作心态 | Be able to correctly understand and evaluate one's own role and maintain a normal working mentality |
|  |  |  |

**护士职业认同量表：**该量表为刘玲等编制，用于测评护士的职业认同水平。共包含30个条目，5个维度，即职业认知评价（9个条目）、职业社会支持（6个条目）、职业社交技能（6个条目）、职业挫折应对（6个条目）和职业自我反思（3个条目）。采用Likert 5级评分法，从“非常不符合”到“非常符合”依次计1～5分。总分30～150分，分数越高提示职业认同水平越高。 该量表已被证明具有较好的信效度，结果显示，该量表的Cronbach's α系数为0.891。已提供护士职业认同条目级记分，护士职业认同维度级记分两个表格。中英文对照如下：

**Nurse Professional Identity Scale**:This scale was developed by Liu Ling et al. to assess the level of professional identity among nurses. It consists of 30 items across 5 dimensions, namely Professional Cognitive Evaluation (9 items), Professional Social Support (6 items), Professional Social Skills (6 items), Professional Frustration Coping (6 items), and Professional Self-Reflection (3 items).

A 5-point Likert scale is adopted for scoring, where responses range from "Strongly Disagree" (scored as 1) to "Strongly Agree" (scored as 5). The total score ranges from 30 to 150, with a higher score indicating a higher level of professional identity.

The scale has been proven to have good reliability and validity, with a Cronbach’s α coefficient of 0.891. Two scoring tables are provided: one for item-level scoring of nurse professional identity and another for dimension-level scoring.The Chinese-English comparison is as follows:

| 序号 | 中文条目 | English translation |
| --- | --- | --- |
| **职业认知评价（Professional Cognition Evaluation, PCE）——9条** | | |
| 1 | 护理工作可让我有价值感 | Nursing allows me to feel a sense of self-worth |
| 2 | 我深信人有主体选择性和能动性，不仅能适应职业环境，还能创造和优化职业环境 | I firmly believe that human beings have subjective selectivity and agency; we can not only adapt to the professional environment, but also create and optimize it |
| 3 | 护理工作与我的兴趣、性格较匹配 | Nursing is well-aligned with my interests and personality |
| 4 | 护士职业可使我更容易赢得社会大众的尊重 | The nursing profession enables me to gain the respect of the public more easily |
| 5 | 离开护士职业会给我造成一些情感创伤 | Leaving the nursing profession would cause me certain emotional distress |
| 6 | 从事护士职业令我愉快 | Working as a nurse brings me joy |
| 7 | 护理工作可使我施展个人的能力和特长 | Nursing allows me to exert my personal abilities and strengths |
| 8 | 我有明确的职业发展目标 | I have clear career development goals |
| 9 | 我感到对继续从事护士职业有种责任感 | I feel a sense of responsibility to continue working as a nurse |
| **职业社会支持（Professional Social Support, PSS）——6条** | | |
| 10 | 同事的理解与支持可使我获得幸福感 | Understanding and support from colleagues can bring me a sense of well-being |
| 11 | 医生和管理者的认可可使我获得幸福感 | Recognition from doctors and administrators can bring me a sense of well-being |
| 12 | 我感到个体可在职业生涯中得到锻炼和成长 | I feel that individuals can temper themselves and grow in their careers |
| 13 | 护理工作可使我和家人握有宝贵的医疗资源 | Nursing provides my family and me with valuable medical resources |
| 14 | 患者和家属的满意评价可使我获得幸福感 | Satisfactory feedback from patients and their families can bring me a sense of well-being |
| 15 | 目前的岗位可使我发挥自身能力，获得幸福感 | My current position allows me to leverage my abilities and achieve a sense of well-being |
| **职业社交技能（Professional Social Skills, PSSk）——6条** | | |
| 16 | 我感到有把握和我遇到的任何一个人接近并打交道 | I feel confident in approaching and interacting with anyone I meet |
| 17 | 我喜欢与人交流，热衷于寻找社交接触的机会 | I enjoy communicating with others and am eager to seek social interaction opportunities |
| 18 | 我每天的工作中充满了令我感兴趣的事情 | My daily work is filled with things that interest me |
| 19 | 我对自己的社交举止很自信 | I am confident in my social demeanor |
| 20 | 面对一个难题时，我会找好友倾诉、听音乐或参加体育活动等调整自己 | When I am in a bad mood, I will talk to close friends, listen to music or take part in sports activities to adjust myself |
| 21 | 我更愿与强过自己的人比较，以激励自己 | I prefer to compare myself with those who are better than me to motivate myself |
| **职业挫折应对（Professional Frustration Coping, PFC）——6条** | | |
| 22 | 职业生涯中遇到困难时，我会积极正面思考，努力寻找解决办法，不轻言放弃 | When encountering difficulties in my career, I will think positively, strive to find solutions, and never give up easily |
| 23 | 职业生涯中遇到挫折时，我不把它当绊脚石，将其视为攀登更高 | When facing setbacks in my career, I do not regard them as stumbling blocks, but as a ladder to climb to greater heights |
| 24 | 心情不好时，我会找好友倾诉、听音乐或参加体育活动等调整自己 | When I am in a bad mood, I will talk to close friends, listen to music or take part in sports activities to adjust myself |
| 25 | 如果我尽量去做，总是能解决问题 | If I do my utmost, I can always solve the problem |
| 26 | 即使别人反对我，我仍有办法取得我所要的 | Even if others oppose me, I still have ways to get what I want |
| 27 | 遇到困难时，我会与比我更困难的人比较 | When facing difficulties, I will compare myself with those who are in more difficult situations |
| **职业自我反思（Professional Self-Reflection, PSR）——3条** | | |
| 28 | 我认为择业既有必然性也有偶然性，你认可它，才会有所作为 | I believe that career choice involves both inevitability and contingency; only when you recognize and embrace it can you achieve something |
| 29 | 我以为专心侍奉自己所从事职业的人，一定能从职业生涯中获得丰厚回馈 | I think that those who dedicate themselves wholeheartedly to their profession will surely reap substantial rewards from their careers |
| 30 | 我感到常分析自己的优势和劣势，可缩小职业生涯中理想与现实自我的差距 | I feel that regularly analyzing my own strengths and weaknesses can narrow the gap between my ideal self and real self in my career |
